# Case Report: An Adolescent Soft Tissue Sarcoma With YWHAE-NUTM2B Fusion Is Effectively Treated With Combined Therapy of Epirubicin and Anlotinib

**DOI:** 10.3389/fonc.2022.905994

**Published:** 2022-06-23

**Authors:** Jiajia He, Yanjie Xu, Xuefeng Ni, Dachuan Zhang, Jiemin Zhao

**Affiliations:** ^1^ Department of Oncology, First People’s Hospital of Changzhou, Changzhou, China; ^2^ Department of Pathology, First People’s Hospital of Changzhou, Changzhou, China

**Keywords:** soft tissue sarcoma, YWHAE-NUTM2B, anlotinib, adolescent, epirubicin

## Abstract

Soft tissue sarcoma is a relatively rare entity that comprises heterogeneous types of tumors. Here we report the case of a 14-year-old girl with pelvic sarcoma with a YWHAE-NUTM2B fusion gene. This fusion transcript has been reported in endometrial stromal sarcomas and clear cell renal sarcomas, but its description in pelvic sarcomas is recent. To our knowledge, this is the first case report describing this translocation in an adolescent patient with soft tissue sarcoma. The patient underwent cytoreductive surgery, followed by systemic chemotherapy and targeted drug treatment. Surprisingly, the treatment was effective, and the young patient is being followed up in our department.

## Introduction

Soft tissue sarcomas (STSs) are a heterogeneous group of malignant tumors arising from mesenchymal cells, which are proportionally more common in children and adolescents (1). The high heterogeneity renders the diagnosis challenging for the pathologists, but molecular analysis can be helpful. With the continuous development of molecular technologies, important refinements in the classification and recognition of new sarcoma entities have been made (2). Notable improvements in diagnosis using novel diagnostic immunohistochemical (IHC) markers and molecular methods have been achieved due to the identification of genetic events across the spectrum of mesenchymal tumorigenesis (3).

In general, treatment for sarcoma patients involves surgery, chemotherapy, radiotherapy, or a combination thereof. For metastatic or advanced STS patients, doxorubicin alone or combined with other cytotoxic drugs has been traditionally recommended as the first-line treatment in the last four decades (4). A deep understanding of the oncogenic pathways and how best to combine targeted agents and/or chemotherapy, and immunotherapy, will be paramount to improve sarcoma prognosis.

This clinical case reports the discovery and management of an adolescent presenting abdominal and pelvic sarcoma with a YWHAE-NUTM2B fusion gene, which has been previously described in clear cell sarcoma of the kidney (CCSK) and high-grade endometrial stromal sarcoma (HGESS) (5, 6). It has been suggested that YWHAE-NUTM2B/E transcript positivity might be associated with higher tumor cellularity, higher disease stage, aggressive clinical behavior, and poor prognosis (7).

Reviewing the literature, we found very few cases of sarcoma with a YWHAE-NUTM2B fusion gene in adolescents, and we have not identified any clear treatment strategy. The aim of the case report is to draw attention to the diagnosis and clinical treatment of this rare but severe sarcoma entity. Our case is interesting for three reasons: the young age of the patient, the extremely progressive development of the tumor, and the effective treatment of combined chemotherapy of epirubicin with anlotinib.

## Case Report

A 14-year-old girl was admitted on September 18, 2021, to the Department of Obstetrics and Gynecology, the Third Affiliated Hospital of Soochow University, Changzhou, China, for moderate, persistent pelvic diffuse pain in order to establish therapeutic specialist conduct. She is a non-smoker and has no history of contraceptive use, has irregular and moderate menstrual cycles, has occasional dysmenorrhea, has no other significant diseases, and has no important medical family history. A huge mass was accessible, reaching the xiphoid process superiorly, and the uterine adnexa was poorly palpated by pelvic examination. Ultrasound suggested a huge abdominal mass. A CT scan of the whole abdomen suggested a huge space occupying the abdominopelvic cavity, blurred surrounding intestinal fat spaces, retroperitoneum multiple enlarged lymph nodes, and unclear double adnexal areas. The tumor marker carbohydrate antigen (CA) 125 was 920.20 U/ml. After informed consent was obtained, exploratory surgery through laparotomy was performed under general anesthesia in our hospital. Specifically, she had right adnexectomy, omentectomy, peritoneal lesion resection, mesenteric lesion resection, and pelvic adhesiolysis. The pelvic cavity was filled with lobulated sarcomatoid tissue, with no obvious envelope. A lobulated sarcomatoid tumor attachment measuring approximately 15 × 15 × 6 cm was seen on the left lateral peritoneal surface. The surface of the omentum was full of lobulated sarcomatoid tissue and measured about 30 × 20 × 20 cm in size. Lobulated sarcomatoid tissue, approximately 8 × 7 × 6 cm to 6 × 5 × 4 cm in size, was found on the intestinal surface and intraperitoneally on the right side of the pelvic cavity, respectively. The uterus was posteriorly positioned, of normal size, smooth, and mobile. The fallopian tube of the left ovary was of normal size and has a smooth surface. The right ovary was slightly enlarged, with edema of the surface and a sarcomatoid tissue attachment on the surface. During the operation, the right ovary was cut, and it was seen that the right ovary was full of sarcomatoid tissue, and the right fallopian tube showed no abnormalities. Scattered masses of about 6 × 5 × 5 cm to 4 × 3 × 3 cm in size were found in the uterine rectal fossa and rectal surface with irregular morphology. The liver surface was smooth, there were no masses in the stomach and diaphragm, and the retroperitoneal lymph nodes were widely enlarged. Postoperative pathology showed neoplastic lesions (right appendix), which tended to be malignant; the type was difficult to determine. No tumor involvement was seen in the ipsilateral fallopian tube. Abdominal wall nodule, intestinal surface nodule, pelvic nodule, greater omentum nodule, and pelvic mass were considered malignant tumors, while the specific type was difficult to determine.

**Figure 1:**
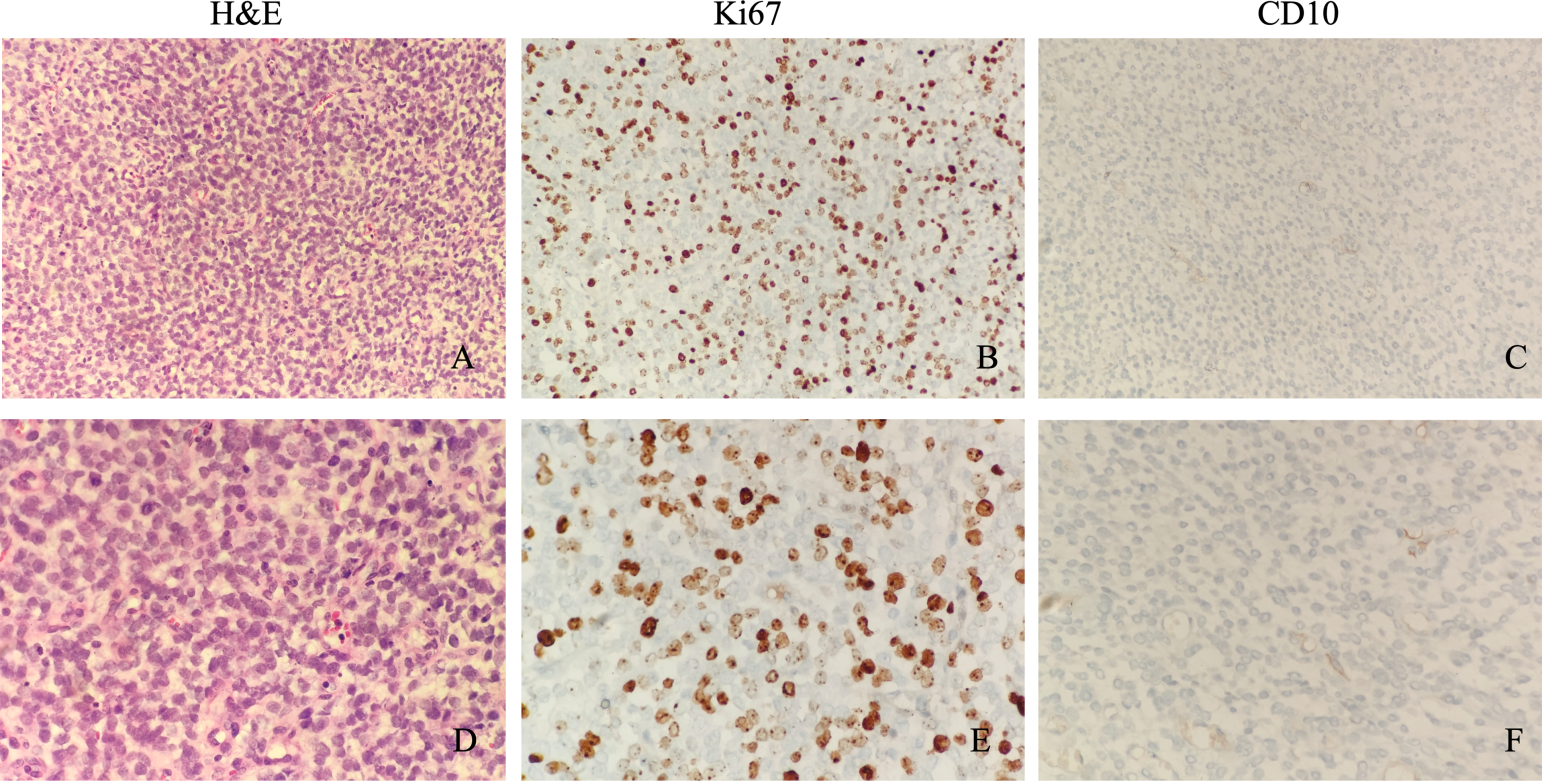
Microscopic appearance of sarcomas with YWHAE-NUTM2B. Microscopically, these tumor cells are made of small round cells, with scanty eosinophilic cytoplasm and a hyperchromatic nucleus (**A**, ×200 magnification; **D**, ×400 magnification). Immunostaining of Ki67 shows a high proliferative index (**B**, ×200 magnification; **E**, ×400 magnification). CD10 was only positive in a small focus of tumor cells (**C**, ×200 magnification; **F**, ×400 magnification).

H&E shows these tumor cells are made of small round cells, with scanty eosinophilic cytoplasm and a hyperchromatic nucleus (**Figures 1A, D**). IHC examination shows positive Ki67 in approximately 60% of tumor cells (**Figures 1B, E**). CD10 was only positive in a small focus of tumor cells, which is a sensitive marker for endometrial stromal sarcoma (**Figures 1C, F**). PAX-8, a marker for serous cell carcinoma, endometrioid cell carcinoma, and clear cell carcinoma of the ovary, showed multifocal/focal positivity. CD99, a marker for Ewing sarcoma, a primitive neuroectodermal tumor, showed diffuse positivity. The immunostaining of the tumor cells was negative for pan-cytokeratin AE1/AE3, CK5/6, CAM5.2, Desmin, EMA, WT1, CgA, Syn, CR, HMBE-1, Inhibin-α, SALL4, and SF-1. The pathological tissues were sent to Fudan University Shanghai Cancer Center, with the following final diagnosis: YWHAE-NUTM2B fusion-positive sarcoma and the molecular pathology (soft tissue next-generation sequencing (NGS)) YWHAE (nm_006761.4: Exon5)-NUTM2B (nm_001278495: Exon2).

**Figure 2:**
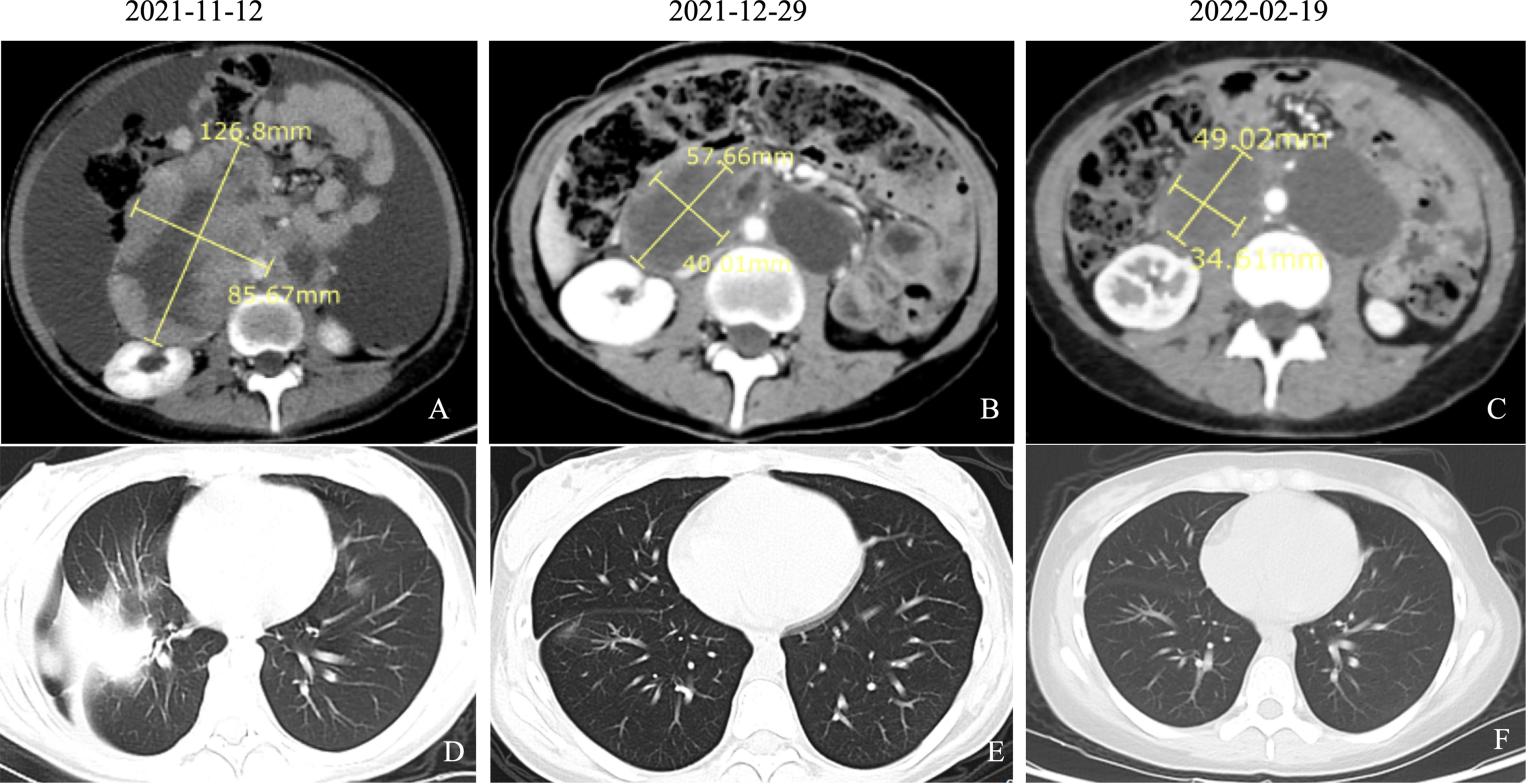
CT findings of pelvic sarcomas and lung metastasis over the course of patient’s treatment. **(A)** Abdominal CT scan (November 12, 2021) showed abdominal and pelvic lesions, blurred surrounding intestinal fat space, and multiple enlarged lymph nodes behind the peritoneum, which were similar to the previous findings (October 20, 2021). Abdominal and pelvic effusions were more advanced than before. **(B)** Abdominal CT scan (December 29, 2021) showed abdominopelvic space occupation, blurred surrounding intestinal fat space, multiple enlarged lymph nodes in the retroperitoneum, and lesions smaller than previously observed (November 12, 2021). **(C)** Abdominal CT scan (February 19, 2022) showed abdominopelvic multiple occupancy, blurring of surrounding intestinal fat spaces, multiple enlarged lymph nodes in the retroperitoneum, and partial reduction of previous (December 29, 2021) lesions. **(D)** Chest CT scan (November 12, 2021) showed right pleural effusion with atelectasis, right pulmonary nodule, and possible metastasis. **(E)** Chest CT scan (December 29, 2021) showed the right pulmonary nodules disappeared compared to the previous ones, and new nodules were observed (possible metastasis). **(F)** Chest CT scan (February 19, 2022) showed the right pulmonary nodule disappeared compared with the anterior part.

On November 11, the patient was taken by cart to our department. On physical examination, she was forced into position, with pale skin, blood pressure 124/106 mmHg, and pulse of 140 beats/min. Multiple enlarged lymph nodes were palpable over the clavicles bilaterally and were fused, qualitatively rigid, and fixed. The abdomen was extremely distended, and a 20-cm surgical scar was seen on the abdomen. Both lower limbs showed serious pitting edema. The laboratory blood tests showed red blood cell (RBC) count 2.84 × 10^12^/L, hemoglobin 7.1 g/dl, sodium 130.2 mmol/L, chlorine 91.40 mmol/L, albumin 28.6 g/L, CA125 179.40 U/ml, and other blood exams in the normal range. A CT scan (November 12, 2021) showed abdominal and pelvic lesions, blurred surrounding intestinal fat space, and multiple enlarged lymph nodes behind the peritoneum, which were similar to previous findings (October 20, 2021). Abdominal and pelvic effusions were more advanced than before, and the chest CT scan showed right pleural effusion with atelectasis, right pulmonary nodule, and possible metastasis (**Figures 2A, D**). Vertical paracentesis was performed, and a huge amount of hemorrhagic ascites was drained. The patient has advanced progressive sarcoma and presented extreme abdominal distension and pain. After rehydration, correction of water–electrolyte balance disorder, enteral and parenteral nutrition support, and symptomatic treatment, the general situation was better than before.

**Figure 3:**
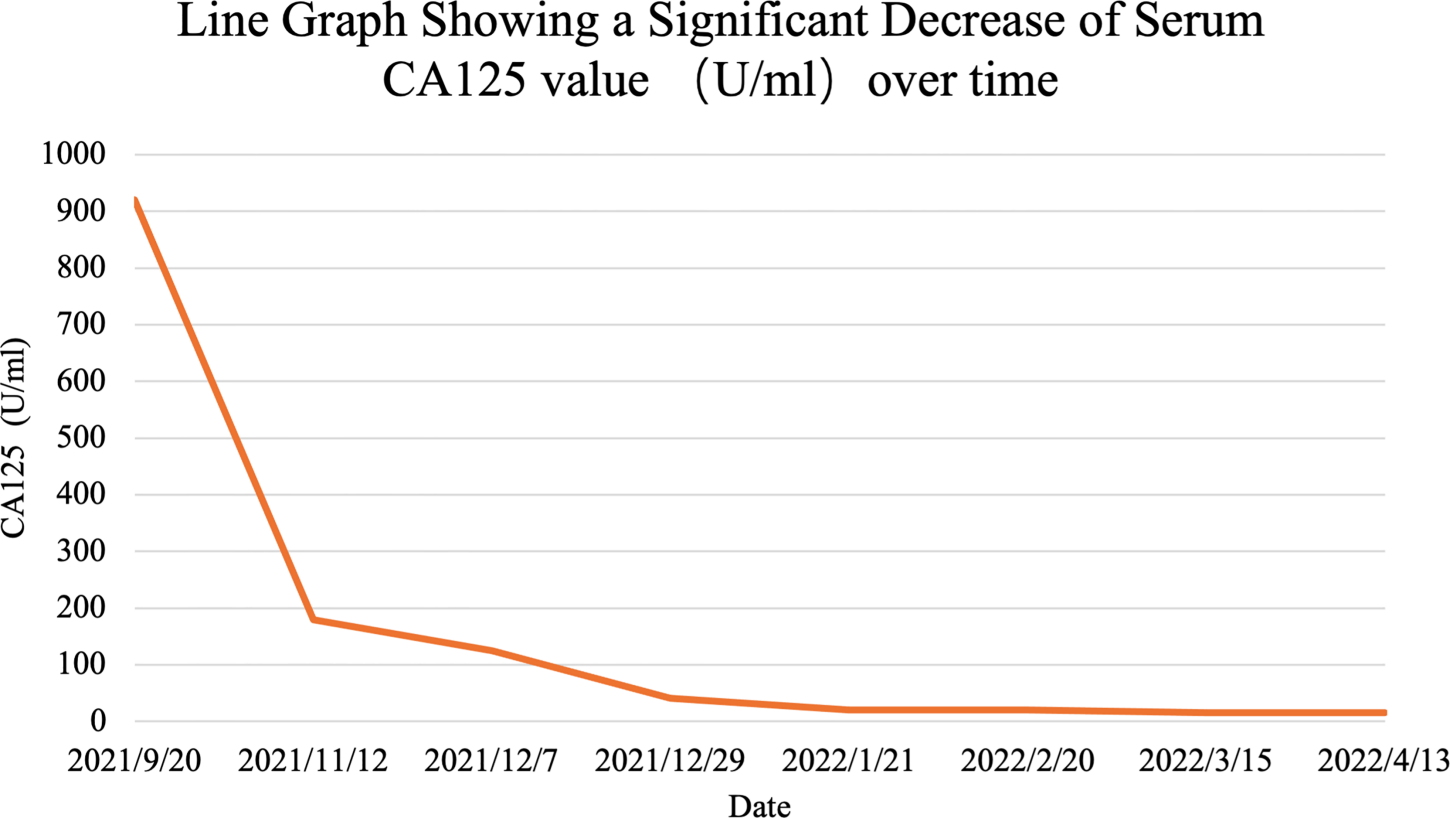
Line graph shows s significant decrease in serum CA125 over the course of patient’s treatment.

Considering the rapid progression of the malignancy, we decided to start the treatment immediately after the improvement of the patient’s general condition. On day 5, she was given antitumor therapy with the consent of her parents. The chemotherapy regimen was as follows: epirubicin 40 mg on days 1 and 2 combined with anlotinib (a small molecular multitarget tyrosine kinase inhibitor) 8 mg/day targeted therapy. The patient was concomitantly treated with suppression of vomiting. The patient’s symptoms of abdominal distension and chest tightness improved after comprehensive treatment. On day 13, the patient was discharged with a better general condition. On November 15, 2021; December 7 and 30, 2021; January 21, 2022; February 21, 2022; March 15, 2022; and April 13, 2022, the patient received epirubicin 40 mg on days 1 and 2 combined with anlotinib 8 mg orally per day. After only 2 courses, the tumor decreased dramatically. CA125, an important ovarian cancer-associated antigen, decreased significantly to 125.40 U/ml as compared to the previous value of 920.20 U/ml. The latest value of CA125 was already decreased to normal (16.0 U/ml) (**Figure 3**). After 4 courses of antitumor therapy, a CT scan (February 19, 2022) showed abdominopelvic space occupation, blurred surrounding intestinal fat space, multiple enlarged lymph nodes in the retroperitoneum, and lesions smaller than previously observed (December 29, 2021) (**Figures 2B, C**). The right pulmonary nodules disappeared as compared with the previous ones, and new nodules were observed (possible metastasis) (**Figures 2E, F**). During the treatment, the patient showed mild leukopenia and mild vomiting, which were improved by recombinant human granulocyte colony-stimulating factor (rhG-CSF) injection and fosaprepitant dimeglumine, respectively.

## Discussion

In the current study, we presented a case of rare pelvic sarcoma harboring the YWHAE-NUTM2B/E fusion transcript in a 14-year-old patient. YWHAE gene belongs to the 14-3-3 family of proteins, which mediate various biological functions, including cell proliferation, differentiation, and metabolism (8). NUTM2B gene belongs to the NUT (nuclear protein in testis) family, which is a relatively unstudied group, consisting of nuclear proteins with poorly described functions. The chromosomal translocation t(10;17) (q22;p13) has been recurrently described in CCSK, which is an uncommon childhood renal tumor that results in a rearrangement of YWHAE on chromosome 17 and NUTM2B/E on chromosome 10 (9, 10). Recently, a neonatal case of round-cell sarcoma with a YWHAE-NUTM2B fusion gene is described, which was very aggressive and led to the death of the infant before the age of 5 months (11). Moreover, the YWHAE-NUTM2 fusion is mostly limited to uterine HGESS except for a few reported cases of uterine angiosarcoma and CIC-rearranged small round cell sarcoma (6, 12). YWHAE-NUTM2A/B fusions have also been described in low-grade ESS, with more aggressiveness and high-grade transformation (13). YWHAE-NUTM2 fusion-positive HGESS was recognized as a new tumor entity and added to the WHO classification in 2014. Due to the rarity of YWHAE-rearranged HGESS, the clinical management of this tumor entity has not yet been fully elucidated.

There are little data about stromal sarcoma’s origin and biology. Histopathological diagnosis is very important for prognosis. However, it is difficult by conventional pathologic means to definitively diagnose soft tissue tumors that require NGS testing. With the wide application of NGS, an increasing number of novel genetic abnormalities in undifferentiated round cell sarcomas have been identified, including BCOR-CCNB3/ZC3H7B/MAML3, CIC-DUX4/FOXO4, YWHAE-NUTM2B gene fusions, or BCOR internal tandem duplication (14). Both YWHAE-NUTM2B fusion and BCOR internal tandem duplication harbor the BCOR overexpression, which could be the oncogenic start point. Soft tissue NGS in the present case showed YWHAE (nm_006761.4: Exon5)-NUTM2B (nm_001278495: Exon2) fusion gene, which belongs to “BCOR rearranged sarcoma.” According to the 5th Edition of the 2020 WHO classification, BCOR rearranged sarcoma is now recognized as a distinct entity due to particular histological characteristics and different clinical outcomes (15). Currently, we cannot decide the tumorigenic origin of the young patient. Gynecologic sarcomas such as endometrial stromal sarcoma could not be entirely excluded since the right ovary was full of sarcomatoid tissue and ovarian cancer-associated antigen CA125 was particularly high.

In the present case, the main and only complaint was abdominopelvic pain; abnormal vaginal bleeding was not recorded. When the patient was admitted to our hospital, her condition was already advanced. Diagnosis of such cases adolescents is not a simple procedure. The tumor mass presented rapid growth, and 34 days after the surgery, the control CT scan showed a huge mass again in the abdominopelvic cavity with repression of the rectum. Due to the lack of knowledge of the fusion transcript, the young patient was diagnosed with “pelvic sarcoma with YWHAE-NUTM2B fusion gene” approximately 1 month after the surgery. It was difficult to confirm the origin of the tumor. There was an urgent need for systematic antitumor therapy due to the rapid growth of the tumor. We therefore developed a therapeutic strategy mainly based on the tumor histology and genetic diagnosis, despite the initial tumor origin.

Due to the extreme rarity of YWHAE-NUTM2B fusion-positive STS in the clinic, currently, no standard therapeutic guidelines are available. The first-line treatment for STS is mainly based on anthracyclines and ifosfamide. Hopefully, there are gradually multiple targeted drugs showing therapeutic effects in STS, bringing clinical benefits to various histotypes of STS (4). Among them, anlotinib, a multitargeted receptor tyrosine kinase inhibitor, has previously shown to have antitumor activity against STS in preclinical and phase I studies, and the toxicity was manageable; therefore, it was approved for the treatment of refractory metastatic STS (16, 17). After 2 courses of combined chemotherapy with epirubicin and targeted drug with anlotinib (epirubicin 60 mg/m^2^ given on days 1 and 2 and anlotinib 8 mg once daily on days 1–14 of each 21-day cycle), the CT showed dramatic tumor regression.

This clinical report leads us to consider not only age and histology in adolescent sarcomas management but also the molecular analyses and genetic diagnosis to develop the therapeutic strategy. Due to the rarity of this pathology, it is difficult to complete randomized clinical trials to determine the optimal management of the tumor.

## Conclusion

STSs are a rare group of tumors that originate from connective tissues. The diagnosis is established by histopathology, extended IHC staining, and molecular tests such as NGS. Rapid diagnosis and timely surgical intervention must be a therapeutic standard in these cases. Traditional chemotherapy for STS is mainly based on anthracyclines and ifosfamide. In front of this complex and highly aggressive YWHAE-NUTM2B fusion-positive STS, we underline its clinical presentation (pelvic primary tumor, aggressive behavior, sensitivity to chemotherapy, and targeted drugs such as anlotinib). We propose the early combined therapy of epirubicin with anlotinib in case of such high-grade sarcomas with Eastern Cooperative Oncology Group (ECOG) scores greater than 2. A meta-analysis from large clinical trials is necessary to determine the prognosis and proper curable treatment of sarcoma with YWHAE-NUTM2B fusion.

## Data Availability Statement

The raw data supporting the conclusions of this article will be made available by the authors, without undue reservation.

## Ethics Statement

Written informed consent was obtained from the individual(s), and minor(s)’ legal guardian/next of kin, for the publication of any potentially identifiable images or data included in this article.

## Author Contributions

JH analyzed the data and drafted the manuscript. JH, YX, and JZ contributed to the conception of the case report. JH and JZ contributed significantly to the analysis and manuscript preparation. DZ helped analyze the pathological data. XN and JZ assisted in drafting the manuscript and critically reviewed the manuscript with regard to oncological issues and constructive discussions. All authors had final approval of the submitted and published versions.

## Conflict of Interest

The authors declare that the research was conducted in the absence of any commercial or financial relationships that could be construed as a potential conflict of interest.

## Publisher’s Note

All claims expressed in this article are solely those of the authors and do not necessarily represent those of their affiliated organizations, or those of the publisher, the editors and the reviewers. Any product that may be evaluated in this article, or claim that may be made by its manufacturer, is not guaranteed or endorsed by the publisher.
